# Feasibility and acceptance of electronic quality of life assessment in general practice: an implementation study

**DOI:** 10.1186/1477-7525-7-51

**Published:** 2009-06-03

**Authors:** Anja Rogausch, Jörg Sigle, Anna Seibert, Sabine Thüring, Michael M Kochen, Wolfgang Himmel

**Affiliations:** 1 Department of Family Medicine, University Medical Center Göttingen, Humboldtallee 38, D-37073 Göttingen, Germany; 2 Institute of Medical Education, Assessment and Evaluation Unit, University of Bern, Konsumstrasse 13, CH-3010 Bern, Switzerland

## Abstract

**Background:**

Patients' health related quality of life (HRQoL) has rarely been systematically monitored in general practice. Electronic tools and practice training might facilitate the routine application of HRQoL questionnaires. Thorough piloting of innovative procedures is strongly recommended before the conduction of large-scale studies. Therefore, we aimed to assess i) the feasibility and acceptance of HRQoL assessment using tablet computers in general practice, ii) the perceived practical utility of HRQoL results and iii) to identify possible barriers hindering wider application of this approach.

**Methods:**

Two HRQoL questionnaires (St. George's Respiratory Questionnaire SGRQ and EORTC QLQ-C30) were electronically presented on portable tablet computers. Wireless network (WLAN) integration into practice computer systems of 14 German general practices with varying infrastructure allowed automatic data exchange and the generation of a printout or a PDF file. General practitioners (GPs) and practice assistants were trained in a 1-hour course, after which they could invite patients with chronic diseases to fill in the electronic questionnaire during their waiting time. We surveyed patients, practice assistants and GPs regarding their acceptance of this tool in semi-structured telephone interviews. The number of assessments, HRQoL results and interview responses were analysed using quantitative and qualitative methods.

**Results:**

Over the course of 1 year, 523 patients filled in the electronic questionnaires (1–5 times; 664 total assessments). On average, results showed specific HRQoL impairments, e.g. with respect to fatigue, pain and sleep disturbances. The number of electronic assessments varied substantially between practices. A total of 280 patients, 27 practice assistants and 17 GPs participated in the telephone interviews. Almost all GPs (16/17 = 94%; 95% CI = 73–99%), most practice assistants (19/27 = 70%; 95% CI = 50–86%) and the majority of patients (240/280 = 86%; 95% CI = 82–91%) indicated that they would welcome the use of electronic HRQoL questionnaires in the future. GPs mentioned availability of local health services (e.g. supportive, physiotherapy) (mean: 9.4 ± 1.0 SD; scale: 1 – 10), sufficient extra time (8.9 ± 1.5) and easy interpretation of HRQoL results (8.6 ± 1.6) as the most important prerequisites for their use. They believed HRQoL assessment facilitated both communication and follow up of patients' conditions. Practice assistants emphasised that this process demonstrated an extra commitment to patient centred care; patients viewed it as a tool, which contributed to the physicians' understanding of their personal condition and circumstances.

**Conclusion:**

This pilot study indicates that electronic HRQoL assessment is technically feasible in general practices. It can provide clinically significant information, which can either be used in the consultation for routine care, or for research purposes. While GPs, practice assistants and patients were generally positive about the electronic procedure, several barriers (e.g. practices' lack of time and routine in HRQoL assessment) need to be overcome to enable broader application of electronic questionnaires in every day medical practice.

## Background

In their Roadmap for Medical Research, the National Institutes of Health (NIH) call for ways to measure patient-reported health-related quality of life (HRQoL) using advanced computer technologies [1]. Comprising physical, social and emotional aspects of patients' well-being, HRQoL is one of the most important patient-oriented outcomes in medical care [2]. Maintenance or enhancement of HRQoL is a relevant therapy goal for patients with chronic (airway) disease in general practice [3]. Systematic HRQoL assessment might facilitate patient management [4,5], the detection of health problems [6-8] and communication between patients and physicians [9] without prolonging encounters. Nevertheless, patients' HRQoL has rarely been systematically monitored on a regular basis, as there are several requirements to be able to optimally utilise this procedure in routine medical care:

• data should be collected completely and accurately with little effort [10],

• data scoring and comparisons to previously collected information should be automated and take place during the office visit [11],

• results should be presented in a user-friendly format, so that patients and physicians can easily understand and discuss them [12],

• results should be assigned to the respective electronic patient record [13] to allow easy monitoring and follow-up over time.

Electronic technology might help to lower the resource burden of HRQoL assessments [14]. A sound implementation of electronic HRQoL questionnaires in general practices includes the following steps: (i) integration of electronic tools into the practice computer infrastructure, which varies from practice to practice, (ii) training of practice assistants and physicians in handling the electronic equipment and interpreting HRQoL scores, and in efficient provision of instructions and information to patients, (iii) continued analysis of any barriers affecting the usability of HRQoL data in daily medical practice and research.

A recent study and our own experience have shown that patients have little difficulty in using a tablet computer [15,16]. Whereas previously published studies were performed in either university-based or hospital settings (in- and out-patient facilities), data we present in this paper expands the focus to include multiple distinct general practices in order to assess acceptance and use of electronic HRQoL questionnaires more generally. Thorough piloting of all procedures concerning complex interventions (such as the implementation of electronic questionnaires into routine care) is recommended before their effect can be studied within larger representative studies [17]. The aim of our study is to implement a tool for electronic HRQoL assessment and to address the following questions:

1. Is it feasible to use tablet computers in the waiting room of general practices to facilitate the routine collection of HRQoL data?

2. Are results from electronic HRQoL assessments, which are immediately available, appreciated by participants and perceived as useful for the consultation and research purposes?

3. What barriers may hinder wider application of this approach?

## Methods

### Setting

This study is part of a primary health care research project ("Medical Care in General Practice"; ) funded by the German Ministry of Education and Research. The research ethics committee of the University of Göttingen approved the study protocol.

### Study population and recruitment

#### Practices

The project was conceptualised as a pilot study with a limited number of participants (15–20 practices). In January 2006, subscribers to a German general practice related e-mail-list were invited to participate in the study (about 60 active subscribers contributed to the mailing list during that particular month). We equipped those practices which gave written informed consent with a portable tablet computer based setup for electronic HRQoL assessment (designated the 'quality-of-life-recorder' or 'QL-recorder'). No specific software or system requirements were necessary for the practice to be eligible for the study. Practice assistants and doctors received small monetary incentives for their participation in the study.

#### Patients

Eligible patients were older than 18 years, suffering from any chronic disease (e.g. osteoporosis, asthma) and able to understand the German language. General practitioners (GPs) and practice assistants were encouraged to invite patients meeting the eligibility criteria at their own discretion (i.e. no obligatory recruitment targets were defined in order to be able to observe the participants' voluntary commitment). Patients' written informed consent was obtained either for the electronic assessment alone or for both the electronic assessment and the telephone interview.

### Instruments and technical procedures

#### Electronic questionnaires

Two questionnaires, the EORTC QLQ-C30 [18] and the St. George's Respiratory Questionnaire SGRQ [19] were electronically displayed on the 'QL-recorder', using a generic electronic questionnaire platform (AnyQuest for Windows) developed by one of the authors [16]. The EORTC QLQ-C30 questionnaire was originally developed to assess the HRQoL of cancer patients but has also been used for patients with various chronic medical conditions, while the SGRQ is specific for patients with chronic airway disease.

For optimal readability and easy usability, the items of the electronic questionnaires were presented in big letters, one item after another. Patients could answer questions by touching the computer screen with an electronic pen, which resembles the handling of a paper-pencil questionnaire. The software ensured that no question was left unanswered unintentionally. Questions that a patient either could not or did not want to answer could be skipped with appropriate documentation. An assessment session could be interrupted at any time and resumed later on.

A movie illustrating electronic HRQoL assessment in general practice is available at .

#### Technical integration

A project member (JS) in collaboration with the practice's system administrator connected the QL-recorder to the practice computer system. Both could be contacted if technical questions arose. The tablet computer could be used anywhere in the practice as the wireless network connection allowed the transmission of patient identification numbers from the practice software to the tablet computer and the return of immediately computed test results to the practice computer system. Depending upon locally established procedures, test results could be imported into the electronic health record, into a specific lab results page, printed, or rendered into a PDF (portable document format) document to be displayed on the doctors' screen or to be added to a paper file as appropriate. The automatically generated cumulative printout included results of previous questionnaire administrations to allow easy assessment of a patient's development over time. "Unfavourable" scores greater than 50 (for EORTC QLQ-C30 function scales: lower than 50) were graphically highlighted on the printout as recommended in a previous study as a rule of thumb [20].

### Training

We developed a 1-hour interactive training course for participating GPs and practice assistants to cover:

1. patient enrolment and obtaining informed consent,

2. an explanation of the handling of the QL-recorder to participating patients,

3. interpretation and use of results during the consultation.

Training sessions took place within participating practices. We provided brief written manuals for the practice staff as well as interpretation aids to be given to patients.

### Data collection

#### Electronic HRQoL assessment

After a short explanation given by the practice assistant, patients could fill in the electronic questionnaire on their own during their waiting time. At the end of the study, electronically collected HRQoL raw data – including number, age and gender of participating patients as well as duration of assessments and test results produced by AnyQuest – were extracted from practice computers and pseudonymised.

#### Telephone interviews

All consenting patients, GPs and practice assistants were interviewed by telephone using semi-standardised interview guidelines. The three guidelines had been developed by a multidisciplinary team, piloted in a pre-study and contained about 10 closed and open questions regarding aspects of the integration of HRQoL assessments into daily routine, possible barriers, perceived benefits as well as sociodemographic data. Participants were asked to rate specific aspects of the HRQoL assessment and then to explain their ratings in an open answer (see example).

Example (physicians' questionnaire):

X1) How do you judge the benefit of electronic quality of life assessment to your practice? Please give a rating between *1 = very good to 6 = insufficient*.

X2) Could you please provide reasons for your answer?

[verbatim transcription of open answers]

Y1) Based on your personal experience, would you welcome the use of electronic quality of life assessment within the daily routine in your practice?

[yes; no; don't know]

Patients were contacted a few days after their initial HRQoL assessment, and GPs and practice assistants after they had conducted at least 5 HRQoL assessments.

At the end of the study period (1 year), GPs were interviewed for a second time and asked to rate different aspects regarding their importance for routine HRQoL assessment (scale 1 = unimportant to 10 = extremely important). GPs who rated the aspect 'financial remuneration' as important (rating minimum = 5) were asked to suggest an adequate amount.

### Data analysis

Answers to open questions were independently analysed and discussed by three researchers (AS, ST, AR) according to the model of inductive category development [21]. Using the software Atlas.ti [22], statements were classified into categories regarding subjective benefits as well as barriers with respect to routine HRQoL assessment. We used a codebook to define resulting categories and anchoring examples. The categories as well as the number of participants who mentioned them are presented in the results section.

Descriptive statistics regarding interview responses, patient characteristics and HRQoL results (frequencies/percentages, 95% confidence intervals CI, means/medians, standard deviations SD and interquartile ranges IQR) were computed using the Statistical Analysis Software package (SAS, Version 9.1).

## Results

### Sample

In response to the invitation, 17 practices (20 GPs) agreed to participate in this study. The practices (8 urban and 9 rural) were spread all over Germany. Three GPs withdrew informed consent later due to personal reasons (severe illness of the practice assistant; change in practice software; lack of time).

**Figure 1:**
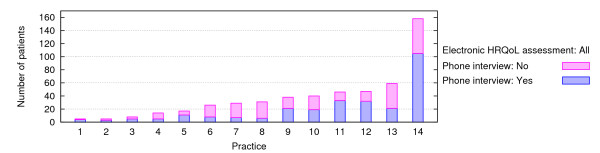
**Number of patients participating i) in the electronic assessment only or ii) both the electronic assessment and telephone interviews**. Bars represent patients per practice participating in the electronic assessment; darker sections indicate patients who additionally participated in the telephone interviews.

According to the practice assistants, virtually all patients who were invited agreed to take part in the study. In total, 523 patients filled in the electronic questionnaires providing 664 assessments (figure 1), with substantial variation between practices (range = 5–205 assessments from 5–158 patients). Out of these, 413 patients completed only one assessment, and 110 patients completed two or more assessments. The total number of follow up assessments was 141, with a maximum of 5 assessments per patient.

A quarter of the patients suffered from a chronic airway disease and consequently answered the SGRQ questionnaire (125/523 patients; 24%); the remainder answered the QLQ-C30. Table 1 shows the characteristics of the 280 patients, 27 practice assistants and 17 GPs who were additionally interviewed by phone. There were no significant differences between the patients who took part in the electronic HRQoL assessment and those who additionally participated in the telephone interviews concerning age (mean: 61 ± 14 SD vs. 62 ± 13 years), diagnosis (chronic airway disease 24% vs. 26%) and distribution of gender (37% vs. 38% male).

**Table 1:** Characteristics of the sample of participants in the telephone interviews.

**Characteristics**	**Physicians** (n = 17)	**Assistants** (n = 27)	**Patients** (n = 280)
**Female**; n (%)	3 (18)	27 (100)	174 (62)

**Age**; mean (± SD)	50 (± 8)	33 (± 12)	62 (± 13)

**Years in (this particular) practice**; mean (± SD)	13 (± 9)	7 (± 6)	13 (± 10)

**Computer literacy**; n (%)			
- skilled user	15 (88)	24 (89)	64 (23)
- some familiarity	2 (12)	3 (11)	45 (16)
- novice	-	-	35 (13)
- none	-	-	134 (48)

**Duration of patients' disease **(years); mean (± SD)	-	-	14 (± 13)

**Severity of patients'disease**; n (%)	-	-	
- minor	-	-	75 (27)
- intermediate	-	-	107 (38)
- serious	-	-	83 (30)
- no information/I don't know	-	-	15 (5)

### Feasibility and results of the electronic HRQoL assessments

The QL-recorders were successfully integrated, typically within half a day, into the 10 different software systems used by the various practices. Rare technical problems could be traced back to instabilities of the wireless networks, but not the QL-recorder itself. No data got lost and results of all HRQoL assessments could be easily exported within a few minutes at the end of the study.

**Figure 2:**
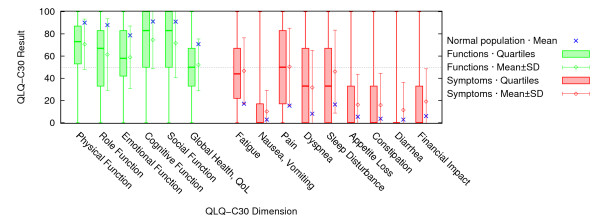
**Results of the initial QLQ-C30 assessment (n = 398 patients)**. For all QLQ-C30 scales, boxplots – including median and interquartile range (box) as well as maximum and minimum (whiskers) – are displayed. Means ± standard deviations from our sample are additionally indicated to facilitate comparisons to mean reference values (asterisks) from the general population [24]. The dotted line represents the "simplified threshold value" of 50; higher values indicate better function (left); lower values indicate lower symptoms (right).

At their initial electronic assessment, patients who completed the QLQ-C30 showed marked impairment compared to the general population in their function scores, global health and symptom scores for fatigue, pain, dyspnea and sleep disorders (figure 2). Similarly, HRQoL of patients with a chronic airway disease was markedly impaired (SGRQ symptoms: median 55.9 [interquartile range IQR 39.6]; activity 53.5 [IQR 43.4]; impact 31.3 [IQR 28.3]; total 39.8 [IQR 30.7]; scale range 0–100 with higher scores indicating more impairment).

### How did GPs, practice assistants and patients evaluate the QL-recorder?

#### Participants' ratings

**Figure 3:**
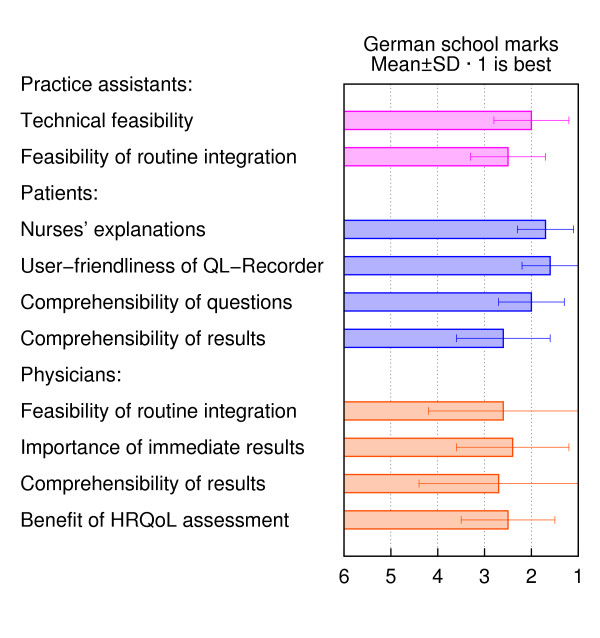
**Evaluation of the HRQoL assessment by participants**.

According to both GPs' and practice assistants' ratings, the HRQoL assessment could be integrated into their daily routine and was useful for patient management (figure 3). Even though half of the patients had little or no experience with computers, they appraised the user-friendliness of the QL-recorder as "good" (mean: 1.6 ± 0.6 SD; scale 1 = very good to 6 = insufficient). About 60% of the patients (165/280) received the printout of their HRQoL results and were, on average, moderately satisfied with its comprehensibility (figure 3).

Practice assistants needed 6 minutes (± 2 min. SD; range 1 – 10 min.) to explain the purpose and handling of the QL-recorder; two-thirds of the practice assistants (67%; 95% confidence interval CI = 46–83%) judged this effort as acceptable (11% found it unacceptable; 22% were undecided). Patients could fill in the electronic questionnaire on their own; on average this required 7 minutes (± 4 min. SD; range 1–37 min.).

Asked whether they felt that the electronic assessment supported their medical care, 192 of 280 patients (69%; 95% CI = 63–75%) agreed (16% disagreed, 15% were undecided). Almost all GPs (16/17 = 94%; 95% CI = 73–99%), most practice assistants (19/27 = 70%; 95% CI = 50–86%) and the majority of patients (240/280 = 86%; 95% CI = 82–91%) indicated that they would welcome the use of electronic HRQoL questionnaires in the future. Patients from practices contributing the highest (> 100), an intermediate (25–100) or a low (< 25) number of assessments differed only slightly with respect to their positive evaluation of the QL-recorder (e.g. 83% [95% CI = 75–89%] vs. 87% [95% CI = 82–92%] vs. 89% [95% CI = 74–97%] of the patients would welcome future HRQoL assessments).

#### Answers to open questions

Patients believed that the HRQoL assessment contributed to the physicians' understanding of their personal condition and circumstances. From their point of view, it helped to focus the consultation, because the GPs were already equipped with information about their current well-being (table 2). GPs recognised the important benefits obtained from the standardised HRQoL information regarding the patients' status, course of disease, and the support for communication – e.g. about sensitive topics. Practice assistants partly referred to the same aspects, but particularly stressed that the HRQoL assessment demonstrated the practice's commitment to patient centred care (table 3).

**Table 2:** Benefits of electronic HRQoL assessment according to patients (n = 280).

Category*	**Example**	Frequency**
Contribution to physicians' understanding of patients' personal condition and circumstances	„The doctor can get a comprehensive overview, because all these different aspects are being asked."	130 (46%)

Focus on patient-physician communication	"If you have answered the questions on the PC, the doctor already knows what to ask in more detail."	114 (41%)

Additional information about current well-being	„The doctor knows me quite well, but it is helpful for him to know how I'm actually doing."	74 (26%)

Information about course of diseases	„If you go to the doctor next time, he can see the changes and compare these to earlier assessments."	73 (26%)

Impulse for self-management	"You can have a look at yourself and think about what you can do by yourself."	60 (21%)

Expression of interest and care	"It makes you feel very sheltered."	50 (18%)

Feedback to adapt treatment	"The doctor gets more information to evaluate the treatment."	47 (17%)

Efficient allocation of resources	"I have time to answer the questions just sitting in the waiting room and the doctor also gains time."	29 (10%)

Information about psychological well-being	"You can figure out better, how one feels inside."	9 (3%)

* as defined according to the qualitative content analysis approach.

** number of patients; mentions of several categories per patients possible

**Table 3:** Benefits of routine HRQoL assessment according to GPs (n = 17) and practice assistants (n = 27).

Category*	**Example**	Frequency**
Focus on patient-physician communication (e.g. on sensitive topics)	„If you see that something is getting worse, it is easier to start talking about the problem"	13 GPs, 3 PA

Information about course of diseases	„The progression over time is most interesting"	11 GPs, 3 PA

Standardised information about current well-being	„It provides comparable results and facilitates documentation"	11 GPs, 1 PA

Contribution to physicians' understanding of patients' personal condition and circumstances	„It gives a holistic view and information, which I otherwise would miss"	9 GPs, 3 PA

Aid for adaptation of medical treatment	„It helps to recognise shortcomings in current therapy"	8 GPs, 2 PA

Commitment to patient centred care	„Patients get the impression of being taken seriously"	6 GPs, 12 PA

Self-reflection and compliance of patients	„Patients can have a look at the results and think about it"	2 GPs, 4 PA

Professionalism and marketing	„It supports the professional appearance of the practice"	5 GPs

Resource management	„You get more information in less time and thus gain time for counselling"	4 GPs

* as defined according to the qualitative content analysis approach.

** number of GPs and practice assistants (PA); mentions of several categories per participant possible

### Structural requirements for routine HRQoL assessment

#### First telephone interview

At the beginning of the study, GPs mentioned a lack of routine and resources as the greatest barriers hindering regular assessments, especially as procedures and HRQoL graphics were unfamiliar (table 4). Practice assistants mentioned 'lack of time' as the main impediment regarding regular HRQoL assessment ('If we have a lot to do, then there is little time for the questionnaire'; 16 practice assistants).

**Table 4:** Barriers regarding routine HRQoL assessment according to GPs (n = 17).

Category*	Example	Frequency**
Lack of practice or routine	„There was a lack of routine or discipline – always to think about it"	13

Lack of time or resources	"We have only one practice assistant and little free time"	13

Unfamiliar graphics	„The results have to be intuitively interpretable at a glance so there is no need for the GP to explain it to the patient"	7

Acute reasons for consultation	„I didn't do it if there was another reason for the consultation, e.g. athlete's foot."	6

Technical problems	"There were sometimes problems concerning the wireless LAN"	6

Undefined consequences	„I didn't know what I should do with the results"	3

Difficulties in understanding (elderly/foreign patients)	"Foreign patients think that they don't understand it"	3

* as defined according to the qualitative content analysis approach.

** number of GPs; mentions of several categories per GP possible

#### Second telephone interview

After having experienced use of the QL-recorder for one year, the participating GPs rated the following as important prerequisites for routine HRQoL assessment: Availability of local health services (e.g. supportive, physiotherapy) (mean: 9.4 ± 1.0 SD; 1 = unimportant, 10 = extremely important), sufficient extra time (8.9 ± 1.5), easy interpretation of HRQoL results (8.6 ± 1.6), immediate availability of results (7.9 ± 2.0), clear responsibility of certain practice assistants for the assessment (6.6 ± 3.2) and financial remuneration (5.6 ± 3.5). On being asked for an estimate regarding appropriate remuneration of electronic HRQoL assessment, GPs recommended compensation of about 12 ± 9 EUR (range 4 – 30 EUR; ≅ 19 USD; 6 – 47 USD) as adequate. Patients' explicit demand for assessments (5.2 ± 3.1), practice advertising (4.5 ± 3.5) and the provision of treatment recommendations based on HRQoL results (3.2 ± 2.9) were regarded less important.

## Discussion

This pilot study describes the implementation of electronic HRQoL assessment in 14 general practices, comprising not only technical integration, but also an on-site practice training session and an evaluation of barriers to its routine use.

### Participation and practice sample

As this was a pilot study, the sample size of practices was limited. Thus, on the practice level, it might be most adequate to interpret the results in a qualitative way. At least three types of responses can be distinguished with respect to the practices: (i) Some subscribers of the mailing list announcing the project may have read the invitation, but decided not to take part. Reasons for non-participation might be limited capacity due to workload or scepticism towards new technologies [23]. (ii) Three practices withdrew informed consent after initially having indicated interest in participation. Reasons for withdrawal included lack of time, change in practice software and severe illness of the practice assistant. Other potential reasons could have been doubt regarding the benefits of electronic HRQoL assessment compared to the effort. (iii) Participating practices were heterogeneous with respect to the GPs' experience, age and gender as well as practice location. This may partly explain the variation in assessment frequencies, which are discussed below in more detail.

Practice assistants reported that virtually all patients who were invited agreed to participate. Hidden decision criteria of practice assistants regarding the selection of patients cannot be ruled out, but were not assessed in the interview. Most patients had little or no experience with computers, and the distribution of age and gender was typical for the general practice population, so we have no clear evidence for a selective invitation, e.g. of younger or more educated patients. Similarly, patients who participated vs. those who did not participate in the telephone interviews showed comparable characteristics.

### Technical feasibility

By means of wirelessly integrated tablet computers, HRQoL data could be easily collected, transferred and automatically printed, making the results available during the same office visit. Thus, several technical and logistic problems such as the patients' inability to handle a mouse or incorrect allocation of patient numbers (IDs) have been successfully solved. Results could be automatically imported into a variety of electronic patient records as recommended by physicians in another study [13]. Patients had no difficulty in completing the HRQoL questionnaires on the tablet computer, which confirms other findings [15].

### The user perspective and utility of results

The majority of participating patients, practice assistants and GPs were satisfied with the electronic HRQoL measurement. GPs appreciated the additional information indicating marked HRQoL impairments in their patients. The assessments showed that most patients had specific limitations e.g. in their physical or role function. Among the QLQ-C30 symptom scales, those for pain, fatigue and sleep disturbance in particular showed clinically significant differences compared to reference values from the general population [24]. These symptoms are often overlooked in daily routine [12]. Asthma patients, too, showed an impaired quality of life in the SGRQ compared to the general population [25], with different patterns in symptoms, activity and the impact of the disease. Results for individual patients showed distinct impairments, rather than uniform patterns, which could help the GP to recognise those patients' individual difficulties.

GPs emphasised that the standardised and reproducible HRQoL results helped them to initiate a focused dialogue with the patient, e.g. regarding sensitive topics. As the questionnaires addressed multiple aspects, patients felt the assessment contributed to the physicians' understanding of their personal condition and circumstances. This is in line with other studies showing that patients perceive HRQoL assessments as a valuable support for their care [26,27] and prefer electronic procedures to paper-pencil assessment [10,28].

### Barriers towards electronic HRQoL assessment

Technically, the HRQoL assessment was functional, well accepted and provided usable HRQoL information. Most participants, however, made less practical use of the new tool than expected. Obviously, there are still barriers to overcome. As indicated by other studies, there seems to be a discrepancy between physicians' appraisal of the importance of HRQoL assessment [29] and the intensity of its application in everyday practice [6,11]. In our study, the HRQoL assessment was organised by the practice staff and took place within the normal routine, while most previous studies employed research assistants to manage the data collection [30].

A typical single-handed German GP may see 50 to 100 patients per day. There are no specialised practice managers, and the practice assistant must complete all administrative and medical tasks per patient within 3 – 15 minutes. In the year of our study, legislative changes increased the practice workload by bringing in new documentation requirements and billing system changes. Germany has the shortest consultation times of several European countries [31]. While practice assistants considered the effort to simply explain the study aims, and the purpose and handling of the QL-recorder acceptable, additional activities – including obtaining formal informed consent – required more time than some practice assistants could afford during busy practice hours. The effort to carry out a HRQoL assessment may be judged positive with the expected benefit in mind, but still be prohibitive given the time pressures of practice reality.

Consistent with this, participating GPs pointed at two primary hindrances: The lack of time to inform patients and to discuss HRQoL data in a busy general practice, and the paucity of resources to alleviate HRQoL deficits. Though the electronic tool reduces workload compared to a paper-pencil measurement, HRQoL assessment still remains an additional task. Time constraints limit the effectiveness of HRQoL assessment if physicians have no capacity to act and appropriately use the information obtained [14,32]. One practice however was able to perform a high number of electronic HRQoL assessments. This practice cared for the population of a larger island, and was run by a GP and practice team with special organisational skills, dedication and proven research interest [33].

### Strengths and limitations

#### Strengths

Our study tried to bring quite advanced tools (HRQoL measurement and up-to-date computer appliances) into multiple, real-life, general practices. Technical function and easy usability demonstrated under these conditions may be considered robust findings, and the transition from a laboratory setting into practice, or from a university clinic into a GP's office, has already taken place. Future clinical trials (e.g. regarding the impact of HRQoL measurement on patient management) can be planned based on the pilot reported here. While it was not the main focus of this study, results of the electronic HRQoL assessment could be further analysed as indicated below.

#### Limitations

The "unprotected" setting of our study meant that our intervention competed with the time required by practice assistants and physicians to carry out established (and essential) procedures. In most practices, our instrument was used less than we had expected. While participants did express their appreciation of HRQoL results in the interviews, we could not examine the consequences of HRQoL measurements, and we have no data regarding objective improvements of care or patients' well-being resulting from the integration of HRQoL assessments into general practices. Due to the methodological approach of a pilot study, including a limited sample size, objective benefits of routine HRQoL assessment, as well as generalisability of the participants' statements, need to be confirmed within a larger controlled study. Ideally information regarding the proportion, motives and characteristics of non-participants should also be systematically collected within these controlled trials.

## Conclusion

The results of this study suggest the following conclusions: (i) electronic assessment of HRQoL data is technically feasible in general practices, (ii) it is welcomed by participants and can provide clinically significant information and indicators to marked HRQoL impairments, which can be useful for clinical or research purposes; (iii) barriers, nevertheless, remain which currently hinder regular HRQoL assessments in general practice.

### Implications for research and practice

The integration of electronic HRQoL assessment into general practices brings with it the prospect of reciprocal transfer of knowledge from patient rated outcomes research into practice and from practice into research. Combining such HRQoL data with information from pseudonymised electronic patient records, (which can be extracted from practice computers after patients' informed consent), would provide a basis for scientific analyses of associations between HRQoL and patients' characteristics, disease and treatment [12]. The availability of HRQoL results immediately during the consultation could contribute to patient centred care, help to focus the patient-physician consultation, support the definition of therapeutic goals as well as the evaluation of their achievement, and provide standardised data, which can be compared intra- and inter- individually.

### Recommendations

To enhance feasibility and usability of electronic HRQoL assessment, we recommend the following steps:

• Training: Though most participants appreciated the 1-hour training, it might be useful to accompany practice assistants during the first days of electronic HRQoL assessment. This could help to bridge the gap between theory and practice, as HRQoL issues have rarely been part of the medical curriculum [34].

• Printout and interpretation: Additional verbal summaries might be easier to understand compared to graphics. As most HRQoL scales did not exceed the threshold of 50 on average, this reference value may be adequate for cancer patients [35] but does not provide sufficient orientation for general practice.

• Adaption to local needs: Practitioners may be interested in selected aspects of HRQoL depending upon their patient clientele or upon the portfolio of supportive measures they can actually provide. For routine care, questionnaires and result presentations should be tailored to these needs to increase the relevance perceived by the GP, and the probability that documented impairments have actual medical consequences.

• Informed consent: Obtaining patients' written informed consent put an extra burden on practice assistants. In order to make the procedure more convenient, data collection would need to be regarded as a standard component of medical service [36], so that written informed consent would not be required for each assessment.

• Incentives: GPs and practice-assistants received only a small financial allowance within this study. Lack of remuneration for HRQoL assessment and discussion of results is regarded as a barrier to its implementation. Also, regular discussion groups of physicians addressing HRQoL topics might be helpful [20], but could not be realised in our project since participating practices were located in distant German regions.

The availability of tools and training is only the first step as the clinical application of HRQoL assessment represents a complex intervention [37]. Critical questions for any larger project in this area are whether the resources for adequate patient invitation and consideration of HRQoL results in medical decision-making can be provided. A thorough understanding of the clinical workflow, practice requirements and goals are essential for the successful implementation of innovative health information technologies in medical practice [38].

## Competing interests

JS has developed the software used to administer questionnaires in this study and provides it as shareware. The remaining authors declare that they have no competing interests.

## Authors' contributions

JS, WH, MK and AR participated in the design, conduction or supervision of the study. AS, ST and AR participated in the acquisition and analysis of the data. JS provided the electronic questionnaires and supported their integration into general practices' infrastructure. AR, WH and JS drafted the manuscript; all authors have been involved in revising the manuscript and gave final approval of this version.
